# Phytochemicals Identification and Bioactive Compounds Estimation of *Artemisia* Species Grown in Saudia Arabia

**DOI:** 10.3390/metabo13030443

**Published:** 2023-03-17

**Authors:** Abdalrhaman M. Salih, Ahmed A. Qahtan, Fahad Al-Qurainy

**Affiliations:** Botany and Microbiology Department, College of Science, King Saud University, P.O. Box 2455, Riyadh 11451, Saudi Arabia

**Keywords:** artemisinin, chromatographical analysis, DPPH, *Artemisia* species

## Abstract

*Artemisia* species are very important medicinal plants, particularly in the Middle East and in developing countries. Their products have been used in traditional and medicine contemporary for the treating of infectious ulcers, gangrenous ulcers, inflammations, and malaria. Artemisinin derived from *Artemisia* species has been used as a drug in many countries for malaria disease treatment. Hence, this study aimed to identify and evaluate the bioactive compounds of three species of *Artemisia* (*Artemisia judaica*, *Artemisia monosperma*, and *Artemisia sieberi*) growing in Saudi Arabia. Therefore, several analytical techniques, such as gas chromatography–mass spectrometry (GC-MS), UV-Visible spectrophotometry (UV-Vis), and high-performance liquid chromatography (HPLC), with reference standards, were used. The GC-MS analysis of the *artemisia* species revealed many bioactive constituents associated with plant secondary metabolites; some of these identified phytochemical components have biological activity. *A. Judaica* showed the highest number of bioactive compounds, followed by *A. sieberi* and *A. monosperma*. Further, the total phenol, total flavonoid, total tannin, terpenoids, and TCA were estimated. Furthermore, biomolecules such gallic acid, tannin acid, quercetin, and artemisinin in different artemisia species were quantified using HPLC with the reference standard. The amount of artemisinin in the leaf extract of these species (*A. sieberi*, *A. Judaica*, and *A. monosperma)* was found to be about 3.01, 2.5, and 1.9 mg/g DW, respectively. Moreover, the antioxidant activity of the samples was estimated. The obtained results have shown that these species possessed high antioxidant activity, and the scavenging of the DPPH radical and hydrogen peroxide were found to be raised with the increase in the plant extract concentration. This reflects the number of bioactive compounds in these species. The findings of this research support and justify the utilization of *Artemisia* species in folk medicine in the Middle East.

## 1. Introduction

*Artemisia* L. is a genus of shrubs and herbs native to temperate Asia (northern temperate regions) [1,2] and it belongs to the Asteraceae family [3], which are mainly found in Asia, North America, and Europe [4]. *Artemisia* species are very important medicinal plants which are currently the subject of bioactive compounds attention because of their cosmetic products, essential oil production, chemical diversity, and biological activity [3,5,6]. For example, the extract of shoots, leaves, and flowers of *Artemisia* species is used traditionally for the treatment of different dieses such as infectious ulcers, inflammations, malaria, and gangrenous ulcers [7,8]. In addition, the oil obtained from *Artemisia* species has been used for hardly curable infectious ulcers treatment and has become famous due to its effects [9], which are insecticidal [10], anticancer [11], antimicrobial [12], and anti-malaria [13,14]. Further, the species of *Artemisia* is being used in the production of cosmetic products extensively [6].

Artemisinin (ART) is one of the most important compound deriveds from *Artemisia* species and has a powerful antimalarial drug with significant activities [15,16]. Additionally, it has been reported that ART as drug has saved the lives of many, particularly in developing countries [17,18]. Further, it has been reported that artemisinin compound and its bioactive derivatives are able to inhibit the metastasis and angiogenesis activity of some cancer cell lines [19].

Antioxidant components such as total phenol, total tannin, and total flavonoid content and their derivatives, such as gallic acid, tannic acid, and quercetin, are considered important nutraceuticals on account of their many health benefits [20]. In the literature, an expanding body of substantial evidence from epidemiological and laboratory studies have proven that some edible plants and their ingredients have antioxidant activities, which have substantial protective impacts on human carcinogenesis [21]. *Artemisia* species possess antioxidant properties which can interfere with oxidative stress to reduce the risk of developing complex diseases [22].

Some species (*Artemisia judaica*, *Artemisia monosperma*, and *Artemisia sieberi*) of the genus *Artemisia* L. are grown in the northern part of Saudi Arabia [23,24]. They are used widely in folk medicine [24] because these species have anthelmintic, anti-inflammatory, antibacterial analgesic, and antipyretic effects [23,24,25]. In addition, documented results showed that the *A. judaica* and *A. sieberi* species grow in Saudia Arabia have anticancer properties [22]. Moreover, these species showed high antimicrobial activities against human pathogens (*Salmonella enteritidis* and *Escherichia coli*) [23]. To our best knowledge, no report was found to be related to the comparative phytochemicals’ studies of these species. Therefore, this work aimed to identify and to estimate the phytochemical compounds of these species. Hence, GC-MS analysis was used for phytochemical constituents screening, a UV-Visible spectrophotometer with reagents was used for antioxidants estimation, whereas HPLC with specific standards was used for the chromatographical separation, identification, and estimation of important bioactive compounds.

## 2. Materials and Methods

### 2.1. Plant Materia Collection

The leaves of three species of *Artemisia* (*A. monosperma*, *A. Judaica*, and *A. sieberi*) were collected during the winter season (January 2021) from the Hail area, the northern part of Saudi Arabia. Voucher specimens (*23396# *A. sieberi*, *23666# *A. monosperma*, and *11413# A. judaica) were deposited in the herbarium (http://www.plantdiversityofsaudiarabia.info/Biodiversity-SaudiArabia/Flora/Flora.htm) (accessed on 1 January 2023) of the Botany and Microbiology Department, King Saud University. The collected leaves were washed well to remove the unwanted particles and dried at room temperature for a further investigation.

### 2.2. Extracts Preparation

The leaves of *Artemisia* species (*A. monosperma*, *Judaica*, and *A. sieberi)* were air-dried and grounded using an electronic blender. Then, 5 g of powdered leaves were extracted in 200 mL of methanol (25 mg/mL) and placed in a shaker at room temperature for 48 h. The leaf extract was filtered using Whatman filter papers No. 1. Next, the extract was allowed to dry naturally at room temperature and stored at 4 °C for further use.

### 2.3. GC-MS Analysis of Leaf Extract

The phytochemical compounds in the leaf extract of *Artemisia* species were identified using GC-MS (Agilent Inc., Palo Alto, CA, USA) coupled to a 5973MSD operated in electron impact mode at 70 eV ion source energy. The gas chromatograph (GC) was fitted with a DB-5MS GC column (30 m length, 0.25 mm inner diameter, and 0.25 µm film thickness). The oven temperature was programmed initially at 40 °C, tracked by a 2 min hold. Then, the temperature was raised to 200 °C at a rate of 5 °C min^−1^. Next, the temperature was raised by 5 °C min^−1^ to 300 °C and held for another 2 min. The injector and detector temperatures were set to 250 and 275 °C, respectively. The total run time of the sample was 40 min. Helium gas was used as the carrier with a flow rate of 1 mL/min and a split flow of 25 mL/min, with the 70 eV electron ionization energy. The detection of phytochemical compounds in the leaf extract was achieved using the matches percentages and commercial libraries of the National Institute of Standards and Technology (WILEY 9th edition, NIST-08 MS library, Gaithersburg, MD, USA).

### 2.4. Estimation of Total Phenolic Content

The total phenolic content (TPC) of the leaf extract was estimated using the Ainsworth [26] method. A volume of 100 µL of the leaf extract was mixed with 100 µL of the Folin–Ciocalteu reagent and 300 µL of sodium carbonate solution (20%). Next, the sample was incubated in the dark for thirty mins at room temperature. The wavelength was recorded at 765 nm using a UV-Visible spectrophotometer (SHIMADZU, UV-1800). The total phenolic in the samples was determined from the following linear equation (y = 0.0021x + 0.0021 with R^2^ = 0.9995) based on a standard curve constructed using different concentrations (25–400 µg/mL) of gallic acid. The total phenolic content was expressed as mg/g DW.

### 2.5. Determination of Total Flavonoid Content

The total flavonoid content (TFC) in plant materials was determined using the method described by Ordonez, A. et al. [27]. A volume of 0.5 mL of methanol extract was mixed with same volume of 2% AlCl_3_ water solution. After 2 h at 25 °C, the wavelength was recorded at 420 nm. The TFC was determined using a calibration curve which is built from different concentrations (50–0400 µg/mL) of quercetin standard with the following equation (y =  0.0172x + 0.0507 with R^2^ = 0.995). The estimated TFC has been expressed as quercetin (mg/g DW).

### 2.6. Determination of Total Tannin Content

The total tannin content (TTC) was calculated from the leaf extract using the method described by Rodrigues et al. [28] with minor changes. A total of 0.1 mL of the extracted samples was added to an Eppendorf tube (2 mL) containing 1.5 mL of Milli-Q water and 0.1 mL of the Folin–Ciocalteu phenol reagent for 8 min. Then, 0.3 mL of 35% sodium carbonate solution was added to the mixture for neutralization. Next, the mixture was shaken well and kept in the dark, at room temperature, for twenty min. The wavelength was recorded at 700 nm. For the determination of the total tannin content in the leaf extract, a calibration curve was constructed using different concentrations of tannic acid standard, and the following equation (Y = 0.0013x+ 0.0052 with R^2^ = 9937) was used. The calculated total tannin content was expressed in terms of mg/g DW.

### 2.7. Estimation of Total Terpenoid

The total terpenoid content in crude extract was estimated using the method described by Batool, R. et al. [29] with light modifications. About 400 mg of powder were taken separately and soaked in 10 mL of absolute ethanol for 24 h. Then, it was filtered and the filtrate was extracted with petroleum ether; the ether extract was treated as the total terpenoid. The total terpenoid content was measured using the following formula: the total terpenoid content (%) = [(final weight of the sample − initial weight of the extract)/weight of the sample] × 100.

### 2.8. DPPH Radical Scavenging Assay

The antioxidant potential of the leaf methanolic extract of *Artemisia* Species (*A. monosperma*, *A. sieberi*, and *A. judaica*) was evaluated using 2,2-diphenyl-1-picrylhydrazyl (DPPH) (Liyana-Pathirana and Shahidi, 2005). In total, 1 mL of the leaf extract at different concentrations (31.25–500 µg/mL) was mixed with the same volume of DPPH (0.135 mM) dissolved in methanol solvent. The mixture was vortexed well and left in the dark at room temperature for thirty min. The optical density of the extract and control (1 mL DPPH + 1 mL methanol) mixture were recorded at 517 nm. The percentage of the DPPH scavenging activity of the extract or standard was estimated using the following formula: DPPH scavenging activity (%) = [(absorbance of control − absorbance of sample)/ absorbance control] × 100, where; absorbance of control is the absorbance of methanol+ DPPH and absorbance of sample is the absorbance of DPPH radical + extract.

### 2.9. H_2_O_2_ Scavenging Assay

The ability of the *Artemisia* leaf extract to hydrogen peroxide (H_2_O_2_) scavenging was estimated according to the method explained by Oyedemi S et al. [30]. In total, 0.1 mL of extracted leaf (31.25–500 µg/mL) was mixed with 0.6 mL of 4 mM of hydrogen peroxide solution prepared in 0.1 M of phosphate buffer (pH 7.4) and it was incubated for 10 min. The reaction mixture was well vortexed. Then, after 10 min of reaction time, the wavelength was recorded at 230 nm. The extract ability to scavenge the hydrogen peroxide was estimated using the following equation: H_2_O_2_ radical scavenging activity = [(absorbance of control − absorbance of sample)/absorbance of control] × 100, where: absorbance of control is the absorbance of methanol + H_2_O_2_ radicals and absorbance of sample is the absorbance of H_2_O_2_ radical + extract.

### 2.10. Determination of Total Antioxidant Activity

The total antioxidant activities (TAC) were measured according to the method described by Prieto et al. [31]. In brief, 0.15 mL of leaf extract was mixed with 1.5 mL of reagent solution that contained 0.6 M of sulfuric acid, 28 mM of sodium phosphate, and 4 mM of ammonium molybdate. Next the reaction mixture was incubated for 90 min at 95 °C. The optical density of the samples was recorded at 695 nm. The total antioxidant capacity was expressed as milligrams of ascorbic acid equivalence (AAE)/g DW.

### 2.11. HPLC Instrument

The Agilent liquid chromatographic system (Agilent Technologies 1290 Infinity system (Agilent Inc., Palo Alto, CA, USA) connected with galaxy (G 4226A) software and the ZOBRAX RX-C18 column (1.8, 4.6 × 150 mm) was used for the separation, identification, and quantification of biomolecules such as artemisinin, quercetin, gallic acid, and tannic acid, with Sigma (Sigma-Aldrich Co., MO, USA) standard for each compound.

### 2.12. Quantification of Gallic Acid

For the gallic acid quantification from the extract using HPLC, the following conditions were followed; 1% aqueous acetic acid solution and methanol in combination of (40:60) (*v*/*v*) used as the mobile phase. The injection volume was 1 µL, the flow rate was 0.700 mL/min, and the temperature of the column was maintained at 25 °C with a 10 min run time. The DAD detector was 274 nm according to the absorption maxima of the analyzed sample. For gallic acid identification, the retention time of the sample was spiked with a standard of gallic acid under similar HPLC conditions, while for the estimation of gallic acid, the following equation (y = 4722.3x − 668.15, R^2^ = 0.968) of the calibration curve, which was constructed from different concentrations (250–1000 µg/mL of gallic acid), was used. 

### 2.13. Artemisinin Quantification

For the separation, identification, and quantification of artemisinin from the leaf extract of *Artemisia* species, the mobile phase consisted of 0.1% phosphate buffer and acetonitrile at a ratio of 70:30 (*v*/*v*). The flow rate was 1.200 mL/min, and the injected volume of the sample was 1 µL with a 5 min run time. The temperature of the column was adjusted at 24 °C. The chromatogram was acquired at a wavelength of 258 nm according to the absorption maxima of the analyzed samples. Sigma Aldrich artemisinin standard was used for the identification of artemisinin in methanolic extract by comparing their retention time under similar conditions. As well, different concentrations (250, 500, and 1000 µg/mL) of artemisinin were used for plotting the calibration curve with the following equation (y = 10.669x − 0.3727, R^2^ = 0.994), which was used for the artemisinin estimation from the methanolic extract.

### 2.14. Quercetin Quantification

The mobile phase used for the separation of quercetin consisted of acetonitrile (A) and methanol (B) (40: 60). The flow rate was 1.00 mL/min, and the injection volume was 1µL with a 10 min run time. The column temperature was maintained at 26 °C. A chromatogram was acquired at a wavelength of 278 nm according to the absorption maxima of the analyzed sample. The quercetin was identified by its retention time spiked with quercetin as a reference standard under similar HPLC conditions. The quercetin in methanolic extract was determined using the linear equation (y = 3245.7x − 61.137, R^2^ = 0.9738) prepared from the different concentrations ((250, 500, 1000 µg/mL) of quercetin standard.

### 2.15. Tannic acid Quantification

For tannic acid quantification using HPLC, the following conditions were used: 0.6% acetic acid solution and methanol at a ratio of 20:80 (*v*/*v*). The injected volume of the sample was 1 µL, and the flow rate was 1 mL/min (361.74 bar) with a 10 min runt time. The temperature of the column was adjusted to 28 °C. The DAD detector was acquired at 274 nm. The tannic acid in the samples was identified by its retention time spiked with the standard of tannic acid under similar HPLC conditions. The tannic acid in the samples was determined using the following equation (y = 964.32x − 52.124, R^2^ = 0.9998) of a standard curve constructed using different concentrations (250–1000 µg/mL) of tannic acid.

### 2.16. Statistical Analysis

The reported data in the tables and figures represent the average of three replicates ± standard deviations (SD). SPSS software, a one way analysis of variance (ANOVA), and Duncan’s test was used for means of the separation and significant level determination at (*p* < 0.05).

## 3. Results and Discussion

### 3.1. GC-MS Analysis of Leaf Extract

In general, plants produce phytochemical constituents as protective mechanisms against abiotic and abiotic stress. These bioactive compounds from medicinal plants are very important in drug manufacturing [32,33]. However, the natural sources of bioactive components have still not been fully discovered, and new sources are really needed. Therefore, the efforts and search for new bioactive products in plants with the hope of discovering new products is an on-going process involving academic and pharmaceutical institutions [34]. The species of *Artemisia* have been playing a vital role in folk as well as contemporary medicine [6]. For the identification of the phytochemical components of *Artemisia* species, samples were subjected to GC-MS analysis. The identification of secondary metabolites from the methanolic extract was done using the matches percentage and commercial libraries of the National Institute of Standards and Technology (NIST). Data in Table 1 show the detected phytochemical compounds form the leaf extract of *Artemisia* species (*A. judaica*, *A. monosperma*, and *A. sieberi*) growing in the northern part of Saudi Arabia, as well as their biological activity. The GC-MS analysis revealed many bioactive compounds related to plant secondary metabolites which possessed anti-allergic, anti-inflammatory, antioxidants, and anti-microbial activity (Table 1 and Figures S1–S4). The variation has been observed in phytochemical compounds among *Artemisia* species; this might be due to the nature and physio-biochemical response of different species to the environmental conditions. It has been reported that various morphogenetic, genetic, and environmental factors can affect the biosynthesis and accumulation of bioactive compounds [35]. Moreover, the production of plant secondary metabolites (antioxidants) depends on many factors such as the plant, its variety, seasonal variations, and environmental conditions, as stated by [36]. According to GC-MS analysis, the species in this genus have different pools of phytochemical components.

### 3.2. Phenolic Compounds Estimation

**Figure 1:**
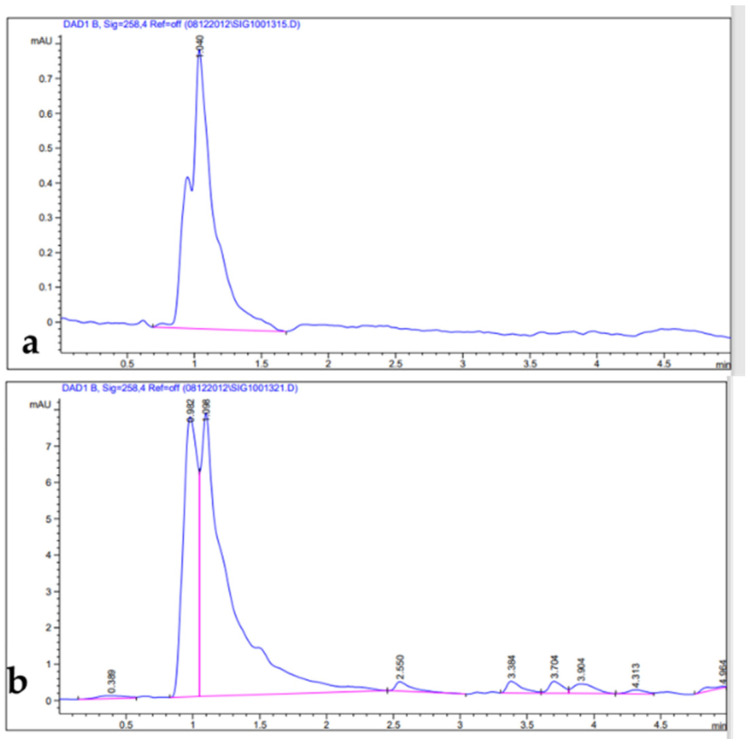
HPLC chromatograms: (**a**) artemisinin standard; (**b**) leaf extract.

**Figure 2:**
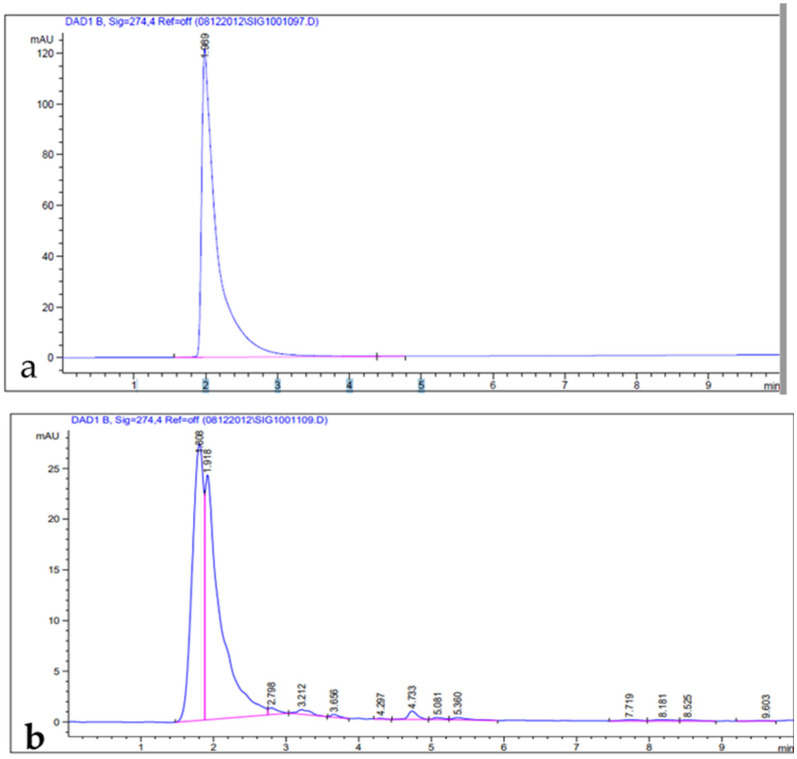
HPLC chromatograms: (**a**) gallic acid standard; (**b**) leaf extract.

**Figure 3:**
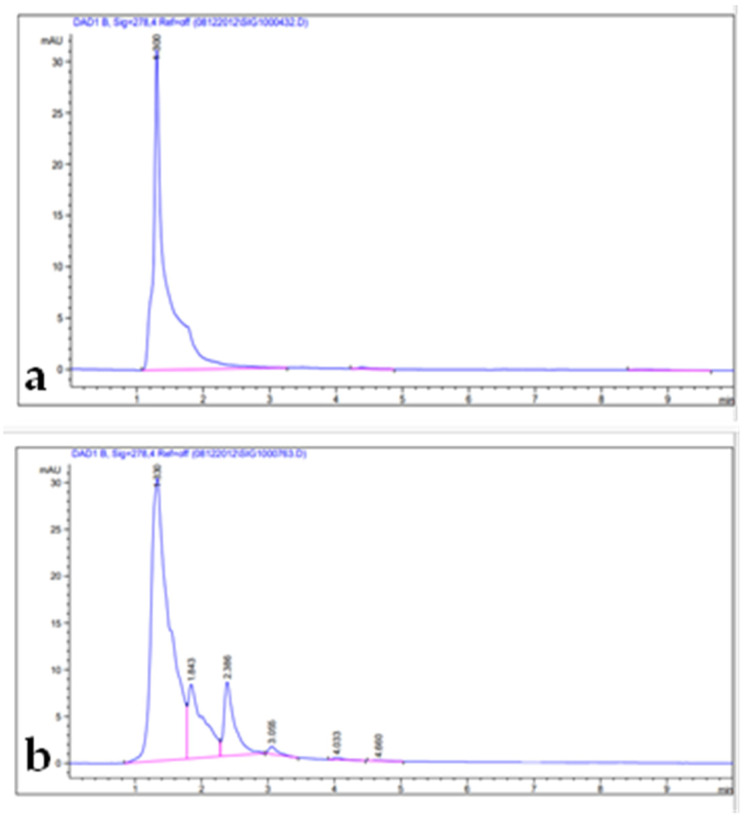
HPLC chromatograms: (**a**) quercetin standard; (**b**) leaf extract.

**Figure 4:**
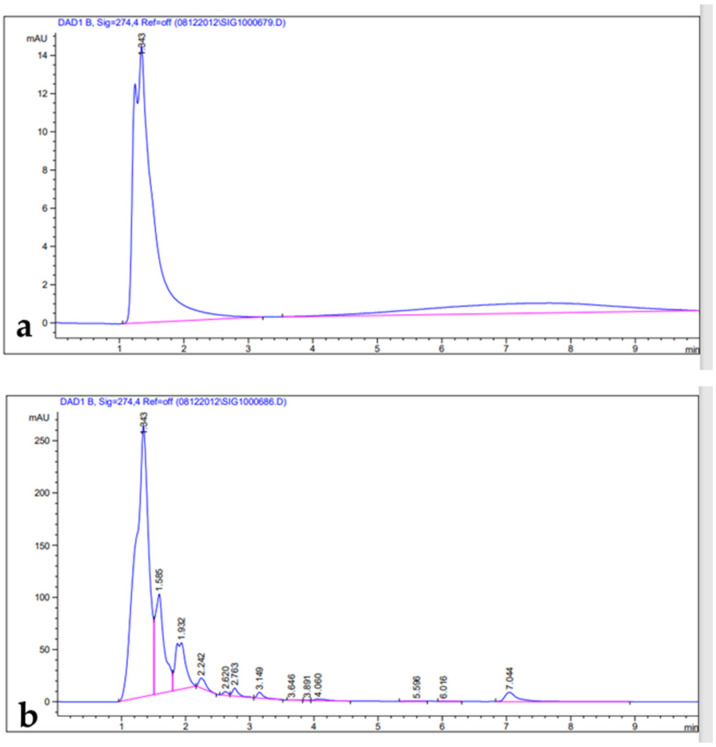
HPLC chromatograms: (**a**) tannic acid standard; (**b**) leaf extract.

Phenolic compounds are well-known as antioxidants components and have many other important uses due to their benefits for human diet, health, and preventing and curing many diseases [51]. Phenolic compounds such phenols and flavonoids are the largest phytochemical constituents with antioxidant properties from plants [52,53]. In this present work, the total phenolic content (TPC), total flavonoid content (TFC), total tannin content (TTC), terpenoids, and total antioxidant capacity (TAC) in the leaves extract of *Artemisia* species were estimated (Table 2). The recorded results showed that the methanolic extract of *A. sieberi* exhibited the highest amount of TPC, followed by *A. judaica* and *A. monosperma*, while *A. Judaica* generated the highest yield of TFC (194.30), tracked by *A. monosperma* (194.30) and *A. sieberi* (120.33 mg/g DW), while *A. sieberi* achieved the highest yield of TTC, followed by *A. Judaica* and *A. monosperma*, as well as the terpenoids result. The highest TCA was recorded by *A. judaica extract*, followed by *A. sieberi* and *A. monosperma* (Table 2). Our findings are consistent with several studies that documented the presence of these constituents in the same species grown in different regions of the world [22,54,55,56,57]. In addition, the leaves of *Artemisia* species contain a higher phenolic acid and flavonoids content, which has health benefits [58]. Furthermore, bioactive compounds such as artemisinin, gallic acid, quercetin, and tannic acid were separated chromatographically and estimated using HPLC with a specific standard for each compound (Figure 1, Figure 2, Figure 3 and Figure 4 and Table 3). The recoded results demonstrated that *A. sieberi* possesses a significant amount of these compounds, followed by *A. judaica* and *A. monosperma.* Although, these biomolecules (quercetin, tannic acid, and gallic acid) are very important; they act as antioxidants as well as have anticancer and antimicrobial activity [59,60,61]. As detailed in this discussion, we focused on artemisinin separation and estimation as an important compound with a potential as antimalarial activity. For example, it has been stated that the derivatives of artemisinin have been used as a drug in many countries to treat malaria disease, particularly in developing countries [62]. For the separation, identification, and estimation of artemisinin from the leaf extract of *Artemisia* species, HPLC with a reference standard (artemisinin) was used. Moreover, mobile phases such acetonitrile, HPLC water, glacial acetic acid, and phosphate buffer with different combinations were tested for the best yield of artemisinin; we found that phosphate buffer and acetonitrile at a ratio of 70:30 was the best mobile phase for artemisinin separation from the samples among different tested solvents, with their conditions detailed in the methodology section. For the estimation of artemisinin from leaf extract, a calibration curve was constructed using different concentrations of artemisinin standard by plotting the area of different concentrations of standard solutions (250–1000 µg/mL). It can be seen from Table 3 that the artemisinin content of the samples ranges from 1.9 to 3.01 mg/g, which illustrated that *A. sieberi* contained the highest amount of artemisinin (3.1 mg/g), while the lowest amount was observed in the leaf extract of *A. monosperma*. This is a very good yield of artemisinin compared to previous reports on the artemisinin product. For example, the previous reports demonstrated that the level of artemisinin in *A. sieberi* was 0.2% of the DW reported by Arab H et al. [63].

### 3.3. Antioxidant Activity Assay

#### DPPH and H_2_O_2_ Estimation

**Figure 5:**
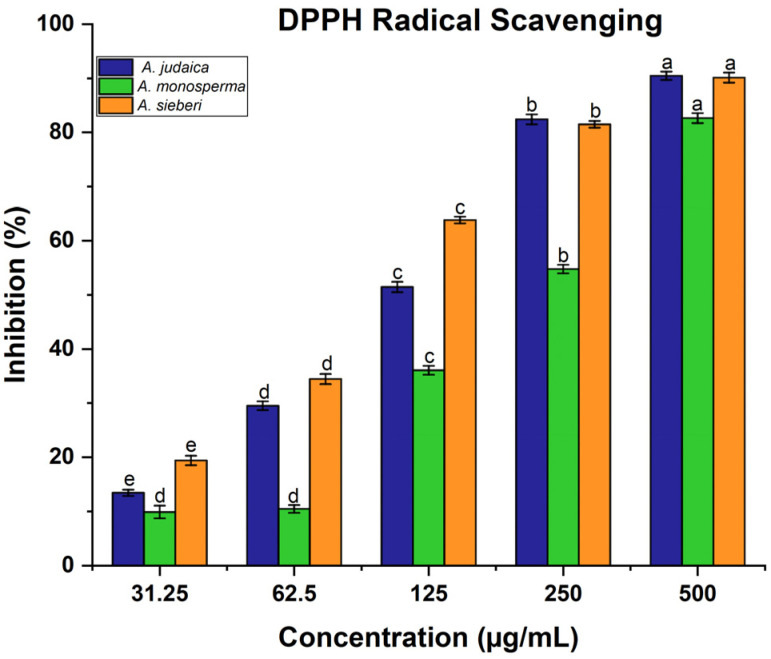
DPPH estimation: the reported data are mean values based on three replicates ± SD. Average in the same column with different letters are significantly different at (*p* < 0.05).

**Figure 6:**
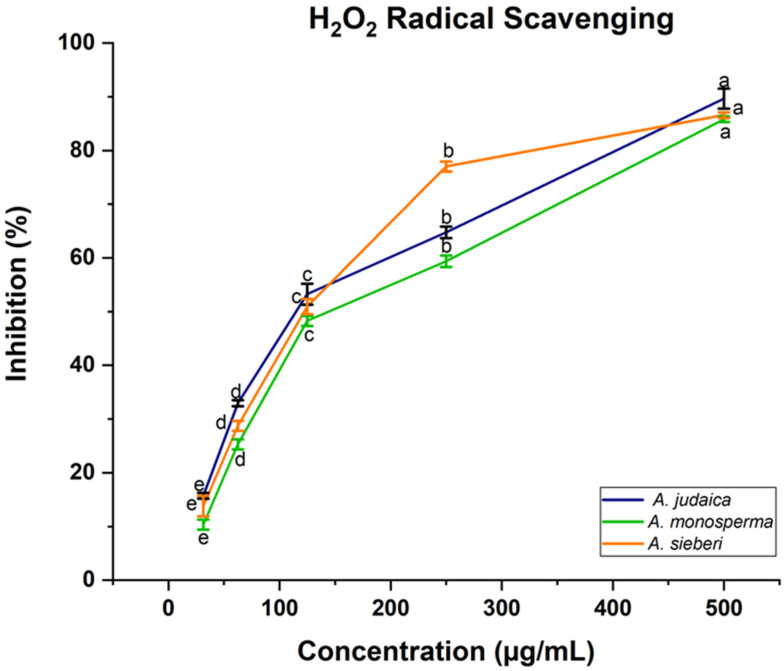
The reported data are average values of three replicates ± standard deviation (SD). ^a,b,c,d,e^ means within the same column with different superscripts are significantly different at (*p <* 0.05).

*Artemisia* species contain high antioxidant activity, which provides considerable protection against may diseases [22,64]. In this present work, the antioxidant activity of the methanolic extract of *Artemisia* species was determined using 2,2-diphenyl-1-picrylhydrazyl (DPPH). The obtained results demonstrated that the scavenging ability of DPPH radical was found to be increased with the increasing of the concentration of leaf extract (Figure 5). It was reported that the antioxidant impact of plant products is mainly due to the radical scavenging properties of phenolic components such as polyphenols, flavonoids, tannins, and phenolic terpenes [65]. For instance, in the literature, expanding reports and evidence from laboratory and epidemiological studies have proven that some edible plants and their components with antioxidant activities have substantial protective impacts on human carcinogenesis [21,66]. Moreover, the ability of the leaf extract of *Artemisia* species to scavenge hydrogen peroxide (H_2_O_2_) was estimated using Oyedemi S. et al.’s [30] method. Likewise, the same result was recorded related to hydrogen peroxide, which showed that the ability of leaf extract was increased with the increase in the leaf extract concentration (Figure 6). The concentration 500 µg/mL was the best of different *Artemisia* species, which is scavenged H_2_O_2_ significantly compared to other concentrations, as shown in Figure 6. For example, in a defense system against pathogenic agents, reactive oxygen species (ROS) induced at low or moderate concentrations have beneficial impacts, and include physiological roles in cellular system responses to anoxia, as reported by Valko M et al. [20]. Our documented results are consistent with previous studies that reported the presence of these compounds in the same species grown in different regions of the world [22].

### 3.4. Qualitative Cluster Analysis and Relationship

**Figure 7:**
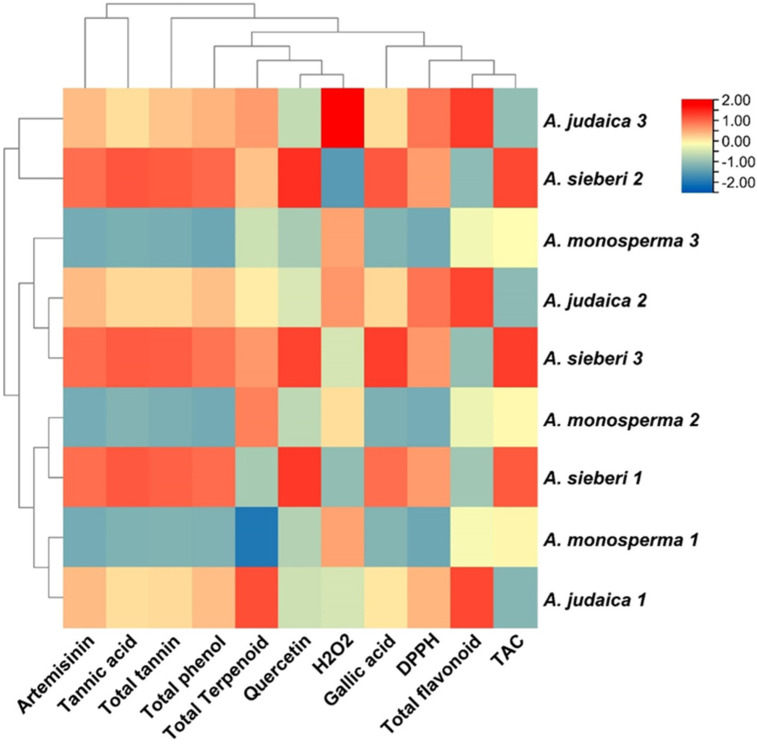
Heat map with cluster of phytochemical constituents from three *Artemisia* species (*A. judaica*, *A. monosperma*, and *A. sieberi*): showed identified levels of phytochemical components according to the colors from red to blue, demonstrated the level of bioactive constituents in decreasing order.

**Figure 8:**
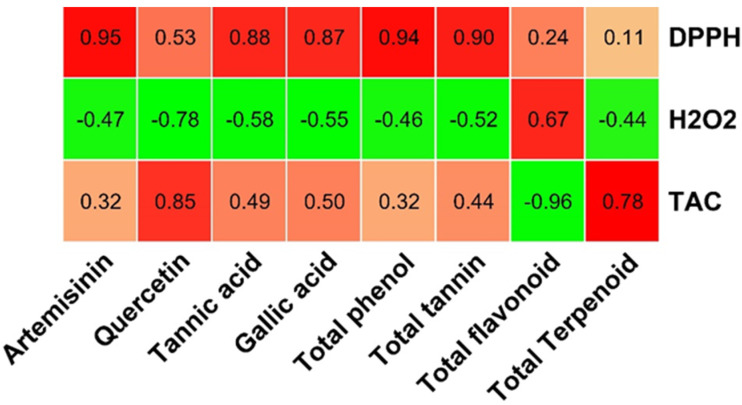
Relation between bioactive compounds and antioxidant assay.

Based on the generated data of the studied parameters, a heat map with cluster analysis was constructed. The dendrogram shown in Figure 7 demonstrated that all the studied parameters can be divided into different clusters; the dendrogram shows the levels of detected phytochemical components according to the colors from red to blue, expressing the level of the compounds in decreasing order. The red color shows a high concentration of bioactive compounds, while the blue color indicates constituents with a low concentration. The relationship between the bioactive compounds and antioxidant activities was investigated. A positive strong relationship was observed between the major compounds (artemisinin, quercetin, tannic acid, gallic acid, total phenol, total tannin, and total flavonoid content with antioxidants assay (DPPH and TCA)) (Figure 8). Plants rich in antioxidant compounds can reduce the pathogenesis and progression associated with oxidative stress [22,67].

## 4. Conclusions

Plant bioactive compounds have been shown to possess several biological effects, which provide scientific evidence for the use of herbs in folk medicine in many ancient communities as well as contemporary medicine. The methanolic extract of *Artemisia* species grown in Saudia Arabia showed many phytochemical compounds, particularly artemisinin, which can be used as a drug for malaria treatment, and the variation in the species was observed. The leaf extract of *A. sieberi* exhibited a higher yield (3.1) of ART, followed by *A. judaica* (2.496), and of *A. monosperma* (2.96 mg/g DW). Our findings confirm the potential use of *A. monosperma*, *A. judaica*, and *A. sieberi* species in traditional medicine and in primary health care. In addition, the obtained findings support the antioxidant properties of *Artemisia* species that can reduce the effect of oxidative stress. Moreover, we concluded that the species of *Artemisia* grown in Saudia Arabia have different pools of bioactive compounds. Further, more investigations in vitro and in vivo should be conducted to evaluate the biological activity of these detected compounds.

## Figures and Tables

**Table 1:** Phytochemical compounds of *Artemisia* species (*A. monosperma*, *A. judaica*, and *A. sieberi*) detected using GC-MS analysis and their biological activity.

S. No.	RT (min)	Name of Compound	Area %			Molecular Weight (g/mol)	Structural Formula	Biological Activity
			A. monosperma	A. judaica	A. sieberi			
1	9.65	Undecane	16.196	1.232	9.235	156.31	C_11_H_24_	Anti-allergic and anti-inflammatory [37]
2	20.102	6-Octen-1-ol, 3,7-dimethyl-, propanoate	10.949	-	-	212.3285	C_13_H_24_O_2_	
3	20.248	(-)-Spathulenol	24.751	-	-	220.356	C_15_H_24_O	Anti-inflammatory and immunomodulatory [38]
4	20.377	Aromadendrene	11.297	-	-	204.3511	C_15_H_24_	Antibacterial [39]
5	20.798	Allylidenecyclohexane	16.392	-	-	122.21	C_9_H_14_	
6	26.312	Hexadecanoic acid, methyl ester	9.352	-	-	270.4507	C_17_H_34_O_2_	Anti-inflammatory, antioxidant, decrease blood cholesterol [40]
7	29.091	9,12,15-Octadecatrienoic acid, (Z, Z, Z)-	11.062	-	-	292.5	C_19_H_32_O_2_	Antimicrobial [41]
8	22.845	Lilac alcohol C	-	1.897	-	170.25	C_10_H_18_O_2_	
9	29.624	2-Methylphenanthro[3,4-d] [1,3] oxazol-10-ol	-	12.230	-	249.26	C_16_H_11_NO_2_	
10	29.779	Etaqualone	-	4.500	-	264.322	C_17_H_16_N_2_O	Anticonvulsant [42]
11	38.664	1,2-Bis(trimethylsilyl)benzene	-	0.366	-	222.47	C_12_H_22_Si_2_	
12	40.092	Cyclotrisiloxane, hexamethyl-	-	1.241	-	222.462	C_6_H_18_O_3_Si_3_	Antimicrobial and antioxidant activities [43]
13	43.095	Tetrasiloxane, decamethyl-	-	34.026	-	310.685	C_10_H_30_O_3_Si_4_	Antifungal activity [44]
14	43.344	Benzo[h]quinoline, 2,4-dimethyl-	-	6.185	-	207.27	C_15_H_13_N	
15	43.903	4-Methyl-2-trimethylsilyloxy-acetophenone	-	20.838	-	222.35	C_12_H_18_O_2_Si	
16	46.165	Silicic acid, diethyl bis(trimethylsilyl) ester	-	17.484	-	296.58	C_10_H_28_O4Si_3_	Antibacterial, antioxidant activity [45]
17	4.472	1-Propanol, 3-bromo-	-	-	6.173	138.991	C_3_H_7_BrO	
18	7.302	Cyclotetrasiloxane, octamethyl-	-	-	4.952	296.6158	C_8_H_24_O_4_Si_4_	Antimicrobial activities [46]
19	9.659	Dodecane	-	-	34.461	170.33	C_12_H_26_	Antibacterial activity [47] and antioxidant [48]
20	14.648	1,3-Cyclopentadiene, 1,2,5,5-tetramethyl-	-	-	5.344	122.2075	C_9_H_14_	Antimicrobial activity [49]
21	14.871	1,3-Cyclopentadiene, 5,5-dimethyl-1-ethyl-	-	-	24.011	122.2075	C_9_H_14_	Antimicrobial activity [49]
22	22.854	Cyclopropane, 1-(1-hydroxy-1-heptyl)-2-methylene-3-pentyl-	-	-	10.903	238.41	C_16_H_30_O	
23	37.271	Benzene, 2-[(tert-butyldimethylsilyl) oxy]-1-isopropyl-4-methyl-	-	-	4.922	264.48	C_16_H_28_OSi	Antibacterial activity [50]

**Table 2:** Total phenol, total flavonoids, total tannin content, terpenoids, and TCA of *Artemisia* species.

Artemisia Species	Phenol (mg GAE/g DW)	Flavonoid (mg QE/g DW)	Tannin (mg TAE/g DW)	Terpenoids (%)	TAC (AAE/g DW)
A. judaica	175.25 ± 0.88 ^b^	24.67 ± 0.07 ^a^	69.24 ± 0.55 ^b^	11.62 ± 0.71 ^ab^	175.91 ± 1.04 ^a^
A. monosperma	120.33 ± 1.53 ^c^	20.39 ± 0.07 ^b^	53.08 ± 0.28 ^c^	10.55 ± 0.72 ^b^	107.33 ± 2.27 ^c^
A. sieberi	194.30 ± 0.84 ^a^	18.22 ± 0.14 ^c^	78.98 ± 0.11 ^a^	13.28 ± 0.41 ^a^	262.08 ± 2.08 ^b^

The data are presented the average of phenol, flavonoid, tannin, terpenoids and TCA in leaf extract of *Artemisia* species ± standard deviation (SD). ^a,b,c^ Means within the same column with different superscripts differ significantly at (*p* < 0.05).

**Table 3:** Bioactive compounds (ART, quercetin, gallic acid, and tannic acid of *Artemisia* species (µg/g)).

Artemisia Species	Artemisinin	Quercetin	Gallic Acid	Tannin Acid
A. judaica	2496.25 ± 0.88 ^b^	15.31 ± 1.17 ^b^	64.29 ± 1.43 ^b^	162.70 ± 1.43 ^b^
A. monosperma	1920.32 ± 1.38 ^c^	10.33 ± 1.19 ^c^	22.144 ± 0.62 ^c^	26.31 ± 1.84 ^c^
A. sieberi	3005.33 ± 1.76 ^a^	58.38 ± 1.67 ^a^	96.79 ± 3.28 ^a^	262.09 ±1.42 ^a^

The data are presented the average of artemisinin, quercetin, gallic acid and tannin acid in leaf extract of *Artemisia* species ± standard deviation (SD). ^a,b,c^ Means within the same column with different superscripts differ significantly at (*p* < 0.05).

## Data Availability

The data presented in this study are available in article and supplementary.

## Supplementary Materials

The following supporting information can be downloaded at: https://www.mdpi.com/article/10.3390/metabo13030443/s1, Figure S1: GC-MS chromatogram of leaf extract of *Artemisia monosperma*, Figure S2: GC-MS chromatogram of leaf extract of *Artemisia sieberi*, Figure S3: GC-MS chromatogram of leaf extract of *Artemisia judaica*, Figure S4: Chemical structures of identified phytochemical compounds from leaf extract of different species (*A. monosperma*, *A. judaica*, and *A. sieberi*) of *Artemisia*.
