# Supplementary material for: Phytochemicals Identification and Bioactive Compounds Estimation of Artemisia Species Grown in Saudia Arabia
Source: Metabolites. 2023 Mar 17;13(3):443. doi: 10.3390/metabo13030443 (PMC10053057; doi:10.3390/metabo13030443)
Supplement: Supplementary file 1 [file metabolites-13-00443-s001.zip › metabolites-2262479-supplementary.pdf]

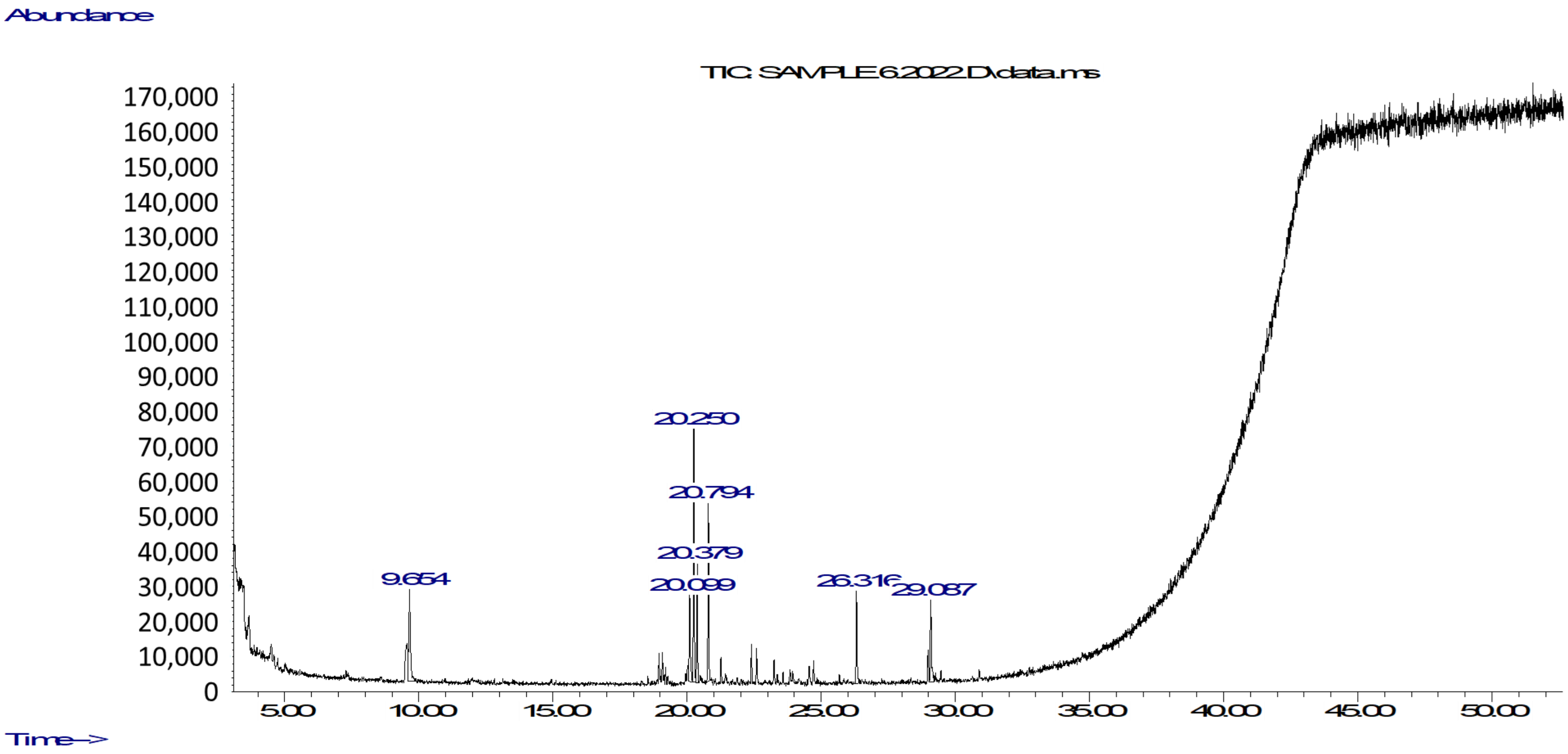

Figure S1. GC-MS chromatogram of leaf extract of *Artemisia monosperma.*

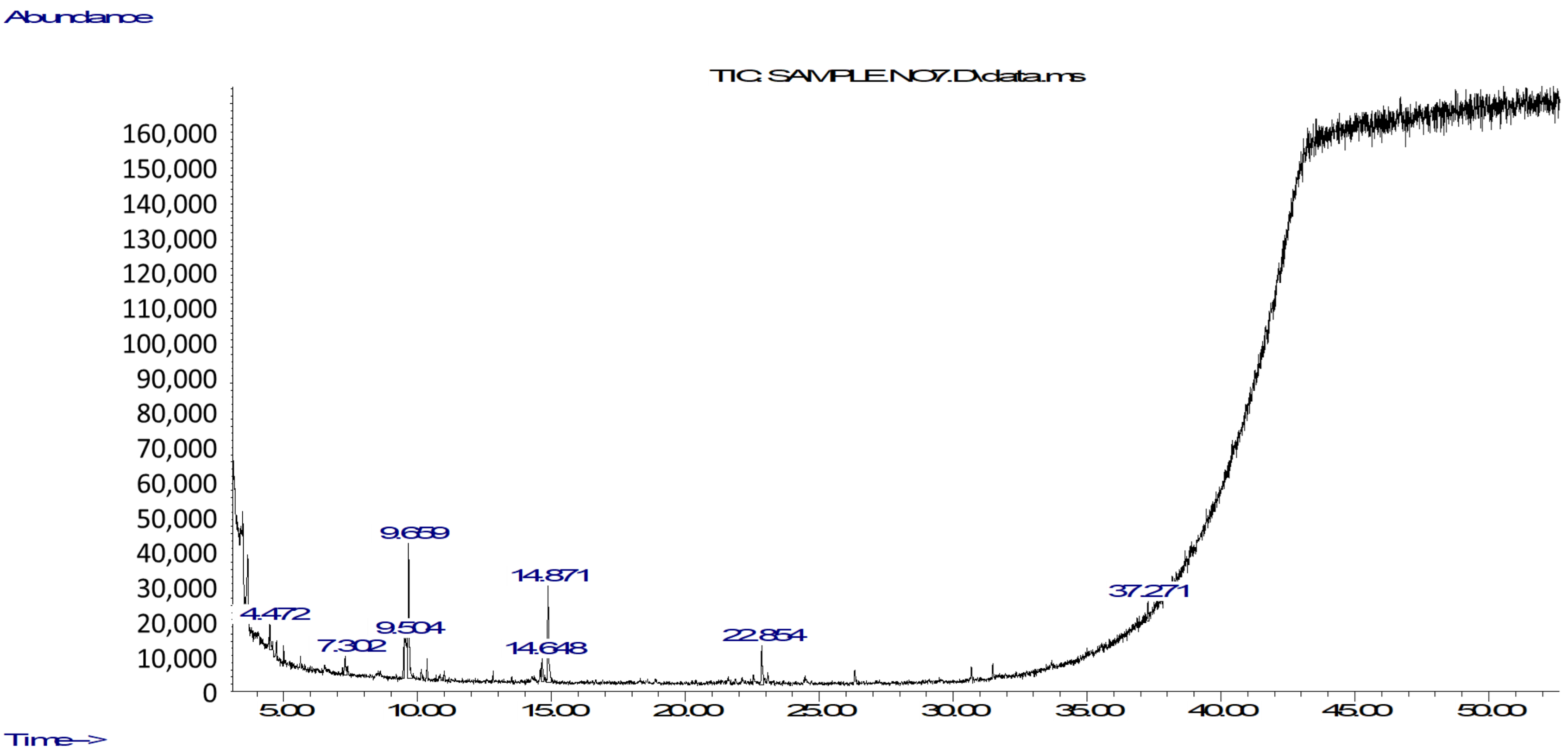

Figure S2. GC-MS chromatogram of leaf extract of *Artemisia sieberi*.

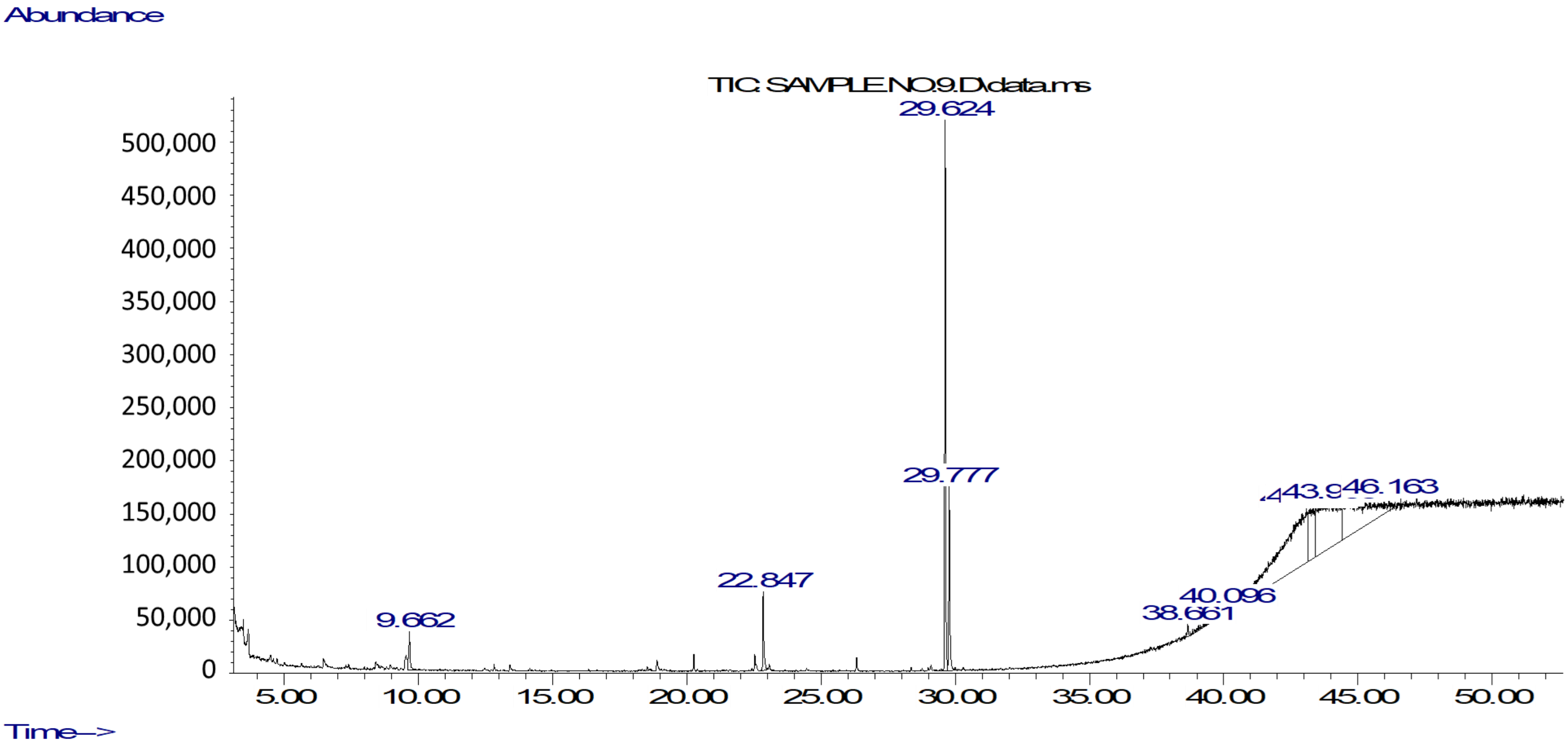

Figure S3. GC-MS chromatogram of leaf extract of *Artemisia judaica*.

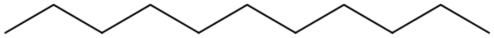

Undecane

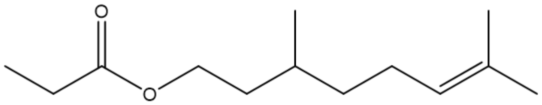

6-Octen-1-ol, 3,7-dimethyl-, propanoate

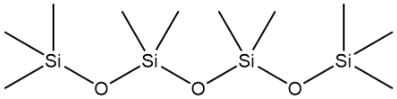

Tetrasiloxane, decamethyl-

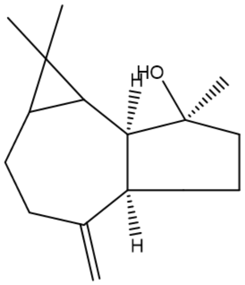

(-)-Spathulenol

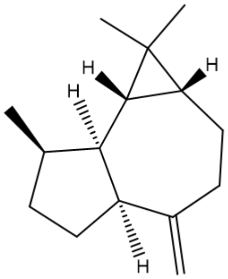

Aromadendrene

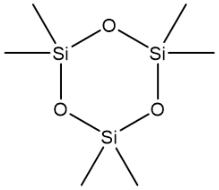

Cyclotrisiloxane, hexamethyl-

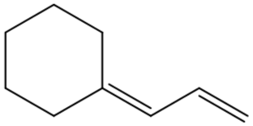

Allylidenecyclohexane

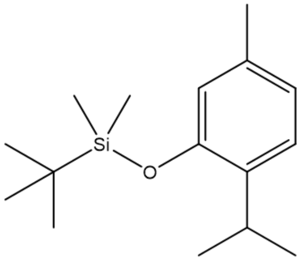

Benzene, 2-[(tert-butyldimethylsilyl) oxy]-1-isopropyl-4-methyl-

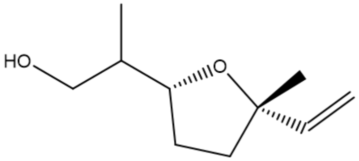

Lilac alcohol C

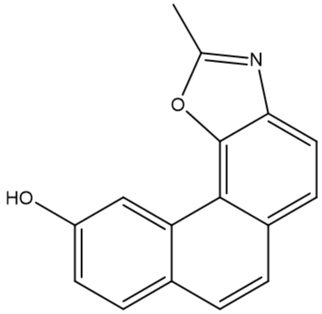

2-Methylphenanthro[3,4-d] [1,3] oxazol-10-ol

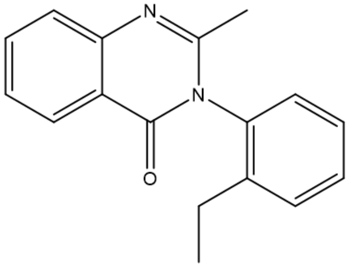

Etaqualone

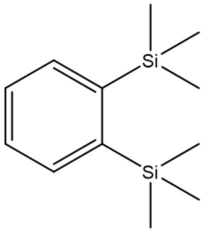

1,2-Bis(trimethylsilyl)benzene

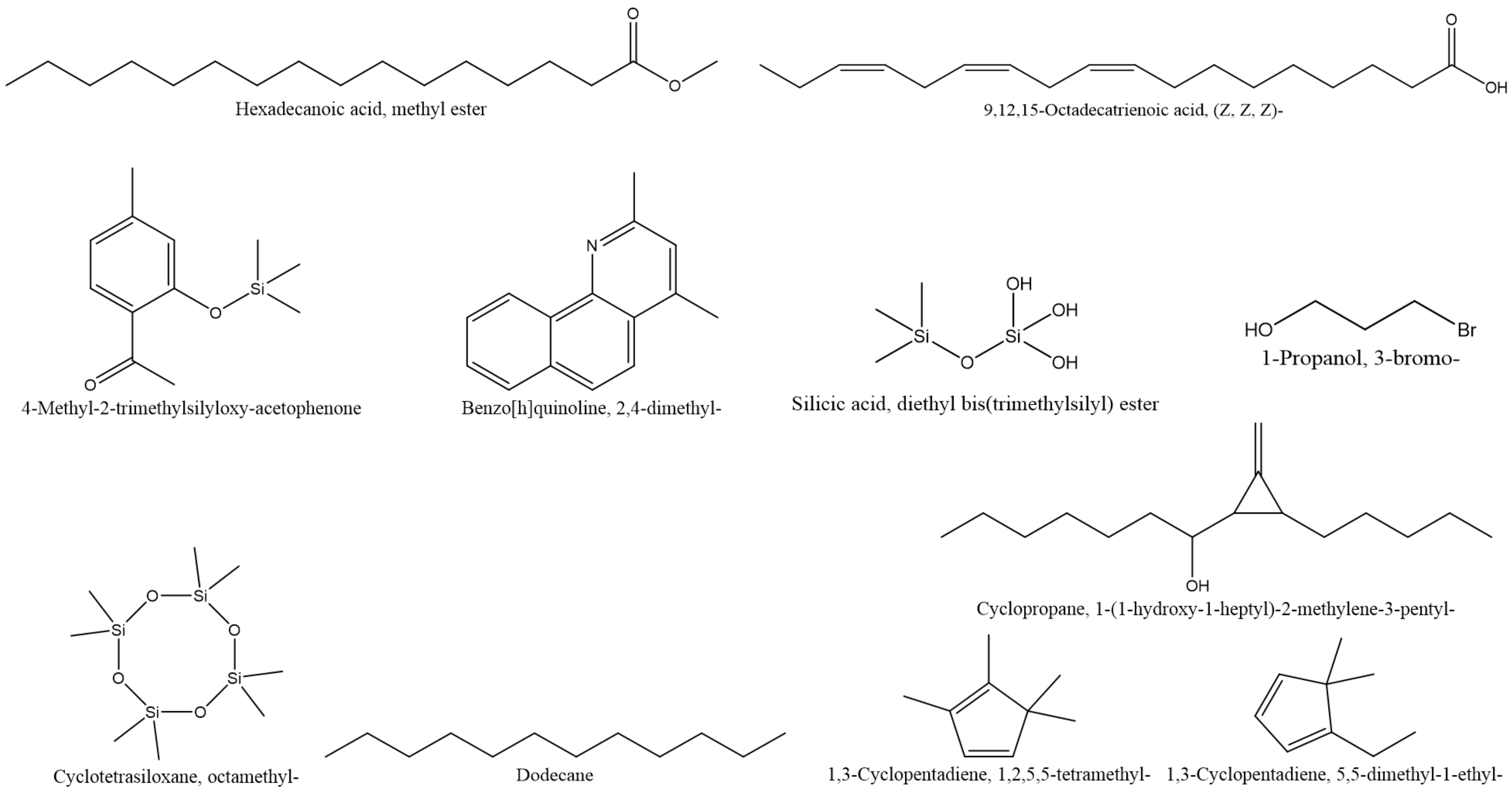

Figure S4. Chemical structures of identified phytochemical compounds from leaf extract of different species (*A. monosperma, A. judaica and A. sieberi*) of *Artemisia.*
